# Prevalence and profile of adverse drug reactions in high-risk pregnancy: a cohort study

**DOI:** 10.1186/s12884-019-2321-8

**Published:** 2019-06-11

**Authors:** Kathlen Dayanne Lopes da Silva, Flávia Evelyn Medeiros Fernandes, Thiago de Lima Pessoa, Sara Iasmin Vieira Cunha Lima, Antônio Gouveia Oliveira, Rand Randall Martins

**Affiliations:** 1 Pharmacy Department, Federal University of Rio Grande do Norte (UFRN), Centro de Ciências da Saúde, Av. General Gustavo Cordeiro de Farias, Petrópolis, Natal, RN 59012-570 Brazil; 2 University Maternity Januário Cicco. Multiprofessional Residency in Health, UFRN, Natal, RN Brazil; 3 Postgraduate Program in Pharmaceutical Sciences, UFRN, Natal, RN Brazil

**Keywords:** Pregnancy, Adverse drug reaction, High-risk pregnancy

## Abstract

**Background:**

**C**ommonly used drugs in pregnant women include antihypertensives, hypoglycemic agents, analgesics, antimicrobials, antiemetics and antispasmodics but the use of medicines during pregnancy, especially in high-risk pregnancy, may be associated with high risk of adverse drug reactions (ADR). The objective of this study was to determine the risk of an adverse drug reaction in hospitalized high-risk pregnant women and the factors associated with their occurrence.

**Methods:**

The study received IRB approval and all patients gave written informed consent. Observational cohort study conducted from September 2015 to November 2016 in 1070 pregnant women consecutively admitted to the high risk sector of the University Maternity Januário Cicco in Brazil. ADR were detected through daily active search. Risk factors for the occurrence of ADR were determined using multivariate logistic regression.

**Results:**

The mean age of the study population was 26.2 ± 7.2 years and gestational age was 31.2 ± 7.2 weeks. The average number of previous pregnancies was 2.4 ± 1.8 and 46.4% reported cases of previous abortion/miscarriage. ADR were observed in 10.7% of women. The main medicines involved, with the incidence rate of ADR per 100 prescriptions of the drug (IR), were parenteral scopolamine (IR 14.9%), methyldopa (IR 15.9%), insulin (IR 8.46%), oral scopolamine (IR 3.58%), captopril (IR 2.38%) and ceftriaxone (IR 18.4%). Multivariate analysis showed that only gestational age in weeks (odds-ratio 0.97, 95% confidence interval 0.95–0.98) was related to the occurrence of adverse reactions.

**Conclusion:**

Lower gestational age is a risk factor for high-risk pregnant women, increasing the likelihood of adverse reactions, with parenteral medications being those that have the highest potential risk of harm.

**Electronic supplementary material:**

The online version of this article (10.1186/s12884-019-2321-8) contains supplementary material, which is available to authorized users.

## Background

High-risk pregnancy is characterized by a high incidence of complications to the mother and/or the fetus during labor or in the postpartum which require specialized care [1]. The frequency of high-risk pregnancy ranges from 25.6 to 63.5% [2–8] with about 216 maternal deaths per 100,000 births [9]. The leading causes of death include cardiovascular disease, preeclampsia or eclampsia, haemorrhage, venous thromboembolism, and amniotic embolism [10]. Non-singleton pregnancies, diabetes mellitus, arterial hypertension and pre-eclampsia [2, 7, 9] are risk factors for complicated pregnancies, especially in the third trimester, and harm to newborns [3]. The third trimester of pregnancy is associated with worsening of pre-existing chronic comorbidities, obstetric complications, and drug administration [11]. Antihypertensive and hypoglycemic agents, analgesics, antimicrobials, antiemetics, antispasmodics, vitamins and minerals are commonly used in pregnancy [12–14], but their use in high-risk pregnant women may be associated with an increased likelihood of adverse drug reactions (ADR). The pharmacokinetics of many drugs are altered during pregnancy due to changes in its parameters, including impaired absorption, increased volume of distribution, increased metabolic rate and changes in renal excretion, and these changes may be more marked in conditions associated with high-risk pregancy. In addition, many drugs have not been evaluated in pregnant women through clinical trials and, therefore, the risk of their use during pregnancy not known [11, 15].

A few studies have estimated the incidence of ADR during pregnancy at around 10% [14, 16, 17], but in general they were based on small samples and the identification of ADR was not through active search. In addition, the drugs involved and the clinical manifestations of ADR have not been adequately described and, to the best of our knowledge, no studies have yet attempted to identify risk factors for the occurrence of ADR. The aim of this study was to estimate, in hospitalized high-risk pregnancy, the risk and type of adverse drug reactions, the drugs more often involved, and the patient factors at admission that may predict their occurrence.

## Methods

Observational, prospective cohort study conducted in the 38-bed pregnancy ward of a maternity school in the city of Natal, RN, Brazil. All high risk patients admitted to the unit between September 2015 and November 2016 were included in this study. Patients were considered high risk if in the investigator’s judgment they presented a clinical condition that if unattended could threaten the life of the mother or the fetus/newborn. Women in whom symptoms of ADR could not be assessed, such as women with cognitive impairment or in coma, were not included. Consent was obtained from a legal representative (first-degree relative) on behalf of participants when they presented impossibility to sign due to difficulty of movement.

In all subjects, data was prospectively collected on clinical variables (age, gestational age, previous pregnancies, abortions and admission diagnosis) and the medications administered throughout the whole length of hospital stay (including the postpartum period). ADRs were defined as any harmful or undesirable and unintentional response occurring with medications at doses normally used in humans for prophylaxis, diagnosis or treatment of a disease, or for modification of physiological functions [18]. The identification and characterization of the ADRs was through active search conducted by two clinical pharmacists: every morning, after the administration of the first daily dose of medication, each patient was questioned about potential discomforts related to the use of drugs. In addition, the clinical charts were checked daily for clinical and laboratory changes that could somehow be related to the prescribed drugs. When an ADR was suspected, both pharmacists applied the Naranjo’s algorithm [19] to assess the causal relationship between the prescribed drugs and the observed clinical manifestations, and the clinical event was classified as definite, probable, possible and doubtful ADR. Only probable and definite ADR were considered in this study.

A sample size of 1070 would afford an estimate of the proportion of women experiencing and ADR with an error of ±1.8%, with 95% confidence, assuming a prevalence of ADR near 10%. The characteristics of the patients are presented descriptively as mean ± standard deviation, or absolute and relative frequencies as appropriate. Drugs involved in ADRs are described as incidence rate of ADRs for every 100 prescriptions of the drug. The identification of risk factors for the occurrence of ADRs was performed through univariate and multivariate logistic regression. Only the variables presenting a *p*-value < 0.20 in univariate analysis were included in the multiple regression model, and for the final model only variables with a p-value < 0.05 were retained. Statistical analysis was performed using Stata 12.0 (Stata Corporation, College Station, TX, USA).

## Results

A total of 1070 women with high-risk pregnancy were included in this study, The mean age of the study population was 26.2 ± 7.3 (range: 16–53) years, the mean gestational age was 31.2 ± 7.2 (range 26–40) weeks, and the average length of stay was 4.4 ± 5.4 (range 1–73) days. The average number of previous pregnancies was 2.4 ± 1.8 and 46.4% women reported previous abortions or miscarriages. The most common admission diagnoses were hypertension (50.2%), preterm labor (27.2%) and gestational diabetes (19.1%). The most often prescribed drugs were nifedipine (38.6%), captopril (31.2%), simethicone (30.2%), scopolamine (28.7%), ferrous sulfate (22.8%) and methyldopa (20.9%) (Table 1). In the postpartum period about 29% (*n* = 310) of the pregnant women returned to the high-risk ward and were monitored.

**Table 1:** Characteristics of the study population (*N* = 1070)

Variables	Values	
Age, years (m, sd)	26.2	7.3
Gestational age, weeks (m, sd)	31.2	7.2
Number of pregnancies (m, sd)	2.4	1.8
Previous abortion/miscarriage (n, %)	496	46.4
Admission diagnosis (n, %)		
Arterial hypertension	536	50.2
Preterm labor	290	27.2
Gestational diabetes	204	19.1
Pyelonephritis	105	9.8
Others	521	57.7
Length of hospitalization, days (m, sd)	4.4	5.4
Number of drugs (m, sd)	4.9	2.5
Drugs (n, %)		
Nifedipine (oral)	413	38.6
Captopril (oral)	334	31.2
Simethicone (oral)	323	30.2
Scopolamine (oral)	307	28.7
Ferrous sulphate (oral)	244	22.8
Methyldopa (oral)	224	20.9

*m* mean, *sd* standard deviation

The proportion of high-risk pregnant women presenting one or more ADRs was 10.7% (114 women, 95% confidence interval (CI): 8.9–12.7%). The description of the observed ADRs is presented in Table 2. The more common ADR were somnolence, blurred vision, nausea, hypoglycemia, dizziness, tachycardia, diarrhea and cough. Uncommon adverse drug reactions, occurring in less than 0.5%, included vomiting, constipation, flushing, drowsiness, phlebitis, dyschromia and abdominal pain. Laboratory changes were rare and included increased urea/creatinine (0.09%) and increased aminotransferases (0.09%).

**Table 2:** Adverse drug reactions observed in a cohort of 1070 high-risk pregnancies

Adverse Drug reaction	number of patients	%
Somnolence	24	2.24
Blurred vision	16	1.50
Nausea	11	1.03
Hypoglycemia	11	1.03
Dizziness	10	0.93
Tachycardia	8	0.75
Diarrhea	8	0.75
Cough	7	0.65
Vomiting	5	0.47
Constipation	5	0.47
Facial flushing	2	0.19
Drowsiness	2	0.19
Increased urea and creatinine	1	0.09
Phlebitis	1	0.09
Increased aminotranferases	1	0.09
Dyschromia	1	0.09
Abdominal pain	1	0.09

Table 3 presents the drugs implicated in ADRs and the estimated incidence of ADR for each 100 prescriptions of the drug. Parenteral scopolamine, methyldopa, insulin NPH, oral scopolamine, captopril and ceftriaxone were the drugs most often implicated, while ceftriaxone, parenteral scopolamine, methyldopa, insulin NPH, and parenteral tramadol are the drugs with higher risk of ADR. All ADR were classified as probable by the Naranjo algorithm.

**Table 3:** Frequency distribution of drugs involved in ADRs and incidence of ADRs per 100 prescriptions of each drug

Drug	Number of ADR	Incidence rate of ADR / 100 prescriptions
Scopolamine (parenteral)	27	14.9
Methyldopa (oral)	18	8.04
Insulin NPH (parenteral)	11	8.46
Scopolamine (oral)	11	3.58
Captopril (oral)	8	2.38
Ceftriaxone (parenteral)	7	18.4
Ferrous sulphate (oral)	5	2.05
Tramadol (parenteral)	3	6.00
Others	24	2.04

Univariate analysis of the association of patient variables at admission with ARD in high-risk pregnancy (Table 4) showed that maternal age (OR 0.98, 95% CI: 0.95–1.01) and gestational age (OR 0.97, 95% CI: 0.94–0.98) were associated with the occurrence of ADR. However, after multivariate analysis only gestational age (adjusted odds ratio: 0.97, 95% CI: 0.94–0.98, *p* = 0.03) was related to the occurrence of ADR.

**Table 4:** Univariate and multivariate analysis of patient variables associated with ADR in high-risk pregnant women

Characteristics	Univariate Analysis				Multivariate Analysis			
	odds ratio	95% CI		*p* value	adjusted odds ratio	95% CI		p value
Age (years)	0.98	0.95	1.01	0.17	0.99	0.96	1.02	0.38
Gestational age (weeks)	0.97	0.94	0.99	0.03	0.97	0.94	0.98	0.03
Number of pregnancies	0.95	0.84	1.08	0.44	–	–	–	–
Previous abortion	0.97	0.67	1.43	0.88	–	–	–	–
Admission diagnosis								
Gestational diabetes	1.21	0.76	1.95	0.42	–	–	–	–
Arterial hypertension	0.95	0.65	1.41	0.81	–	–	–	–
Pyelonephritis	1.45	0.81	2.60	0.21	–	–	–	–

## Discussion

Many studies in the literature discuss ADR in hospitalized patients, however only a few have investigated their occurrence in pregnant women. Our study involved the active search for ADRs in over one thousand high-risk pregnant women observed throughout their stay in a specialized unit over a period of 15 months in a reference maternity school and the main findings of this study were that about 1 out of 9 high-risk pregnancies admitted to a hospital will develop one or more ADRs, that parenteral scopolamine and oral methyldopa are the medications most frequently involved in ADRs, that parenteral drugs such as scopolamine, ceftriaxone and insulin have the greatest risk of ADR. Regardless of the administered drugs, lower gestational age and lower gestational age are likely important predictors of ADR.

Published studies on ADR in pregnancy have shown very different prevalences among them, with estimates ranging between 0.3 and 20.0% [14, 16]. The only Brazilian work on the topic [17] reported that 8.8% women from a sample of 294 high-risk hospitalized pregnant women suffered an ADR. Hernández-Hernández et al. [16], in a Mexican cohort of 207 pregnant women, observed ADR in 12.1%. In contrast, a French study found ADR in only 0.3% of pregnancies, but data on ADR were obtained by self-reporting [20]. A European study evaluating the frequency of medication-related problems in hospitalized pregnant women reported ADR in 9.9% of them [14]. Several other papers were based on rather small samples, on retrospective data collection, or ADR were identified only through self-reporting.

Although the occurrence rate is rather high, ADR in high-risk pregnant women tend to be of low severity. Clinical manifestations such as somnolence, facial flushing and blurred vision are self-limited and usually did not imply changes in pharmacotherapy. Of the adverse reactions detected, only hypoglycemia related to insulin use presents a greater risk to the patient and the need for immediate intervention.

Parenteral scopolamine and oral use of methyldopa were identified as more involved in ADR, a finding similar to that described in another study [19]. The use of scopolamine was more common in postpartum. This drug, because of its anticholinergic action in several organs, has the potential for a wide range of adverse reactions due to an extension of its pharmacodynamic profile resulting in excessive anticholinergic activity [21]. On the other hand, its antimuscarinic action may be potentiated by the association with other drugs with anticholinergic properties, such as antiemetics [22]. In this study, the observed effects correspond to those described in the literature: dizziness, tachycardia, blurred vision, constipation and somnolence. Methyldopa is the drug of choice for gestational hypertension due to the absence of teratogenicity and fetal toxicity. Medications like methyldopa that act on the central nervous system can cause sedation, drowsiness, depression and have a significant effect on psychomotor performance [23]. However, only sedation related to the use of methyldopa was detected in this study. Some anti-hypertensive medicines administered to patients in this study, such as captopril, were prescribed only in the postpartum.

The drugs for parenteral use (ceftriaxone, scopolamine and insulin) presented a greater risk for the development of ADR. A large study that analyzed risk factors for adverse events in hospitalized patients characterized the parenteral route as more implicated the occurrence of ADR [24]. Critical patients also have this characteristic, and intravenous administration is responsible for a 3% increase in the risk of ADR for each drug used [25].

The multivariate model identified only low gestational age as a factor related to the occurrence of ADR in high risk pregnancy. This can be explained because during pregnancy several organ systems are affected by substantial anatomical and physiological changes. Many of these significantly affect the pharmacokinetic (absorption, distribution, metabolism, and elimination) and pharmacodynamic properties of different therapeutic agents [26]. In our results, drugs more involved with ADR have hydrophilic properties (scopolamine, methyldopa, insulin and captopril). The rise of total body water, blood volume, and capillary hydrostatic pressure significantly increases the volume of distribution of hydrophilic substrates; however, these changes stabilize and decrease in the last weeks of pregnancy [27]. Theoretically, lower gestational age would require higher doses of hydrophilic drugs due to hemodilution, thus implying a higher risk of ADR. In contrast, in pregnancy the renal plasma flow increases by 25 to 50% and glomerular filtration rate by 50% and there is an increase in the activity of the organic cation and anion transporters [28]. Therefore, it is unclear whether the ADR were a consequence of pharmacokinetic changes in pregnancy [27].

The study presents as main limitation the collection of data in a single institution. Despite this, some methodological characteristics validate the results, such as the large sample size, the prospective cohort design, and ADR detection through daily active search.

The characterization of the prevalence, medications involved and clinical manifestations of ADR allows the multiprofessional team to better manage the occurrence of these reactions. Information on drug toxicity is essential for the development of strategies that minimize the risk of harm and improve safety in the pharmacotherapy of hospitalized pregnant women. Despite the predominance of ADR of mild severity, new research evaluating potential clinical outcomes associated with the occurrence of ADR in high-risk pregnancy is needed.

## Conclusion

Our study found that ADR in high-risk pregnancy are usually of mild severity but occur in about one tenth of patients, and that lower gestational age increases the risk of their occurrence in this population. Parenteral scopolamine and oral methyldopa are the drugs more often involved in ADRs. However, parenteral drugs such as scopolamine, ceftriaxone and insulin have a greater risk for the development of ADR.

## Additional File

Additional File 1: ADR study database in pregnant women. The database that supports our findings in this study is presented as an additional file. (XLSX 386 kb)

## Abbreviations

ADR: Adverse Drug Reaction
WHO: World Health Organization
